# Phase II multicenter trial combining nivolumab and radiosurgery for NSCLC and RCC brain metastases

**DOI:** 10.1093/noajnl/vdad018

**Published:** 2023-03-01

**Authors:** Philip Wong, Laura Masucci, Marie Florescu, Marc-Emile Plourde, Valerie Panet-Raymond, Michel Pavic, Scott Owen, Laurence Masson-Coté, Cynthia Ménard, Bertrand Routy, Mustapha Tehfe, Kristoff Nelson, Francois Guilbert, Olivier Boucher, Sareh Keshavarzi, Normand Blais, David Roberge

**Affiliations:** Division of Radiation Oncology, Centre Hospitalier de l’Université de Montréal, Montreal, Quebec, Canada; Radiation Medicine Program, Princess Margaret Cancer Centre, University Health Network, Toronto, Ontario, Canada; Division of Radiation Oncology, Centre Hospitalier de l’Université de Montréal, Montreal, Quebec, Canada; Division of Hematology Oncology, Centre Hospitalier de l’Université de Montréal, Montreal, Quebec, Canada; Department of Radiation Oncology, Centre Hospitalier Universitaire de Sherbrooke, Sherbrooke, Quebec, Canada; Department of Radiation Oncology, McGill University Health Centre, Montreal, Quebec, Canada; Department of Hematology Oncology, Centre Hospitalier Universitaire de Sherbrooke, Sherbrooke, Quebec, Canada; Institut de recherche de l’Université de Sherbrooke, Sherbrooke, Quebec, Canada; Department of Medical Oncology, McGill University Health Centre, Montreal, Quebec, Canada; Department of Radiation Oncology, Centre Hospitalier Universitaire de Sherbrooke, Sherbrooke, Quebec, Canada; Division of Radiation Oncology, Centre Hospitalier de l’Université de Montréal, Montreal, Quebec, Canada; Division of Hematology Oncology, Centre Hospitalier de l’Université de Montréal, Montreal, Quebec, Canada; Division of Hematology Oncology, Centre Hospitalier de l’Université de Montréal, Montreal, Quebec, Canada; Department of Radiology, Centre Hospitalier de l’Université de Montréal, Montreal, Quebec, Canada; Department of Radiology, Centre Hospitalier de l’Université de Montréal, Montreal, Quebec, Canada; Department of Psychology, Centre Hospitalier de l’Université de Montréal, Montreal, Quebec, Canada; Department of Biostatistics, Princess Margaret Cancer Centre, University Health Network, Toronto, Ontario, Canada; Biostatistics Division, Dalla Lana School of Public Health, University of Toronto, Toronto, Ontario, Canada; Division of Hematology Oncology, Centre Hospitalier de l’Université de Montréal, Montreal, Quebec, Canada; Division of Radiation Oncology, Centre Hospitalier de l’Université de Montréal, Montreal, Quebec, Canada

**Keywords:** brain metastases, checkpoint inhibitors, lung cancer, radiosurgery, renal cell cancer

## Abstract

**Background:**

Anti-PD-1 has activity in brain metastases (BM). This phase II open labeled non-randomized single arm trial examined the safety and efficacy of combining nivolumab with radiosurgery (SRS) in the treatment of patients with BM from non-small cell lung cancer (NSCLC) and renal cell carcinoma (RCC).

**Methods:**

This was a multicenter trial (NCT02978404) in which patients diagnosed with NSCLC or RCC, having ≤ 10 cc of un-irradiated BM and no prior immunotherapy were eligible. Nivolumab (240 mg or 480 mg IV) was administered for up to 2 years until progression. SRS (15–21 Gy) to all un-irradiated BM was delivered within 14 days after the first dose of nivolumab. The primary endpoint was intracranial progression free survival (iPFS).

**Results:**

Twenty-six patients (22 NSCLC and 4 RCC) were enrolled between August 2017 and January 2020. A median of 3 (1–9) BM were treated with SRS. Median follow-up was 16.0 months (0.43–25.9 months). Two patients developed nivolumab and SRS related grade 3 fatigue. One-year iPFS and OS were 45.2% (95% CI 29.3–69.6%) and 61.3% (95% CI 45.1–83.3%), respectively. Overall response (partial or complete) of SRS treated BM was attained in 14 out of the 20 patients with ≥1 evaluable follow-up MRI. Mean FACT-Br total scores were 90.2 at baseline and improved to 146.2 within 2–4 months (*P* = .0007).

**Conclusions:**

The adverse event profile and FACT-Br assessments suggested that SRS during nivolumab was well tolerated. Upfront SRS with the initiation of anti-PD-1 prolonged the 1-year iPFS and achieved high intracranial control. This combined approach merits validation randomized studies.

**Key Points**

In this phase II trial involving patients with brain metastases, SRS during the 1st cycle of Nivolumab was safe and resulted in high intracranial disease control.
Further study of concurrent immune checkpoint inhibition with SRS is justified.

**Importance of the Study**

Stereotactic radiosurgery (SRS) and anti-PD-1 therapy are independent active treatments against renal cell (RCC) and non-small cell carcinoma (NSCLC) brain metastases (BM). This phase II multicenter trial evaluated the combined use of SRS during the first cycle of anti-PD-1 therapy, nivolumab, for patients with RCC or NSCLC BM. Patient adverse event and quality of life profiles suggested that upfront SRS during nivolumab was well tolerated. Upfront SRS with the initiation of anti-PD-1 resulted in high intracranial control rate that was comparable to historical results of patients treated with whole brain radiotherapy and SRS. Median 1-year intracranial progression free survival was also longer than prior studies in which patients with RCC or NSCLC BM were treated with anti-PD-1 alone. The combined approach of SRS during anti-PD-1 therapy merits validation randomized studies.

It is estimated that 20–40% of patients with cancer will develop brain metastasis (BM) during the course of their illness. Improvements in local and systemic therapies have led to improved patient survival and more opportunities to develop BM. Whole brain radiation therapy (WBRT) was the mainstay of treatment for BM but can lead to persistent fatigue, alopecia and cognitive decline.^1^ Radiosurgery (SRS) is increasingly administered as the sole initial treatment, to spare patients acute and long-term side effects associated with WBRT.^1^ Local control alone of small BM following SRS treatment can approach 90%. However, without adding WBRT, 55–78% of patients will develop new BM following SRS.^2–5^

Cancer immunotherapy has demonstrated clinical efficacy in various cancers. As monotherapy, PD-1 inhibitors have demonstrated activity against brain metastases (BM) from non-small cell lung cancer (NSCLC) and clear-cell renal cell carcinoma (RCC).^6–8^ Median intracranial progression free survival (iPFS) in these studies ranged from 2.3 to 2.7 months.^9–11^ The efficacy of immunotherapy may be further enhanced by combining it with radiotherapy.^12^ The ideal sequence, timing and dose of RT that stimulate the immune system in the presence of immune checkpoint inhibition is currently unclear. We hypothesized that a single highly cytotoxic and accurate dose of SRS given concurrently (within 14 days) with the first Nivolumab administration would stimulate the immune system. This investigator-initiated phase II trial examined the safety and efficacy of combining nivolumab with radiosurgery (SRS) in the treatment of patients with BM from NSCLC and RCC.

## Methods

### Study Design and Participants

This was a multicentre open-label single arm phase II trial in which adult patients (≥18 years of age) diagnosed with NSCLC or RCC, having ≤ 10 cc of un-irradiated BM, no WBRT and no prior immunotherapy were eligible. Other eligibility criteria included Eastern Cooperative Oncology Group performance status of 0–1, ability to complete neurocognitive and quality of life questionnaires (QOL) without assistance, less than 4 prior lines of systemic treatments and adequate hematological, renal and hepatic functions. Exclusion criteria included pregnant or nursing women, BM in the brainstem, patients experiencing seizure within 7 days of registration without the use of corticosteroids, and inability to undergo MRI. Patients requiring systemic treatment with >10 mg prednisone equivalent corticosteroids were not eligible. This study was approved by each institution’s ethics review board and informed patient consent was obtained prior to enrollment. Patients were recruited from three centers in Quebec, Canada. The latest version of the protocol is in the Supplement.

### Study Treatment

Study treatment commenced with nivolumab (either 240 mg or 480 mg IV), which was continued for up to 2 years at bi-weekly or monthly intervals until progression or unacceptable treatment-related toxicities. SRS (15–21 Gy) to all visible un-irradiated brain lesions was administered within 14 days after the first dose of nivolumab (cycle 1). The suggested SRS doses per lesion were 15–20 Gy, 15–18 Gy and 15 Gy in 1 fraction for lesions measuring <2 cm, 2–3 cm and 3–4 cm in maximum diameter, respectively.^13^ As MRI could be repeated for SRS planning, if additional lesions or growths were identified on the radiosurgery planning MRI scan (compared to the diagnostic/screening MRI scan), SRS would be delivered to additional lesions provided that the maximum volume of intracranial disease did not exceed 15 cc at the time of planning MRI and the treating physician deemed the case safe and appropriate for SRS.

At the CHUM and MUHC, SRS was delivered using Cyberknife^®^ (Accuray, Sunnyvale, California). At the CHUS, Gamma Knife^®^ (Elekta AB, Stockholm, Sweden) was used to deliver SRS. In all cases, the PTV margins were 0 mm. Mean conformity index and new conformity index were 0.99 [standard deviation (SD): 0.04] and 1.0 (SD: 0.04), respectively. Accounting for the plans used to treat all of lesions in each patient, the mean brain volume within the 8 and 12 Gy isodose lines were 4.0 cc (SD 4.2 cc) and 8.0 cc (SD 8.9 cc), respectively.

### Assessments

Adverse events (AE) were assessed at every cycle and prior to SRS using the Common Terminology Criteria for Adverse Events (CTCAE) version 4.3. The relationship of AE to nivolumab and/or SRS was established jointly by the treating medical oncologist and radiation oncologist. Nivolumab interruptions were allowed for AEs. Treatments were discontinued if there was disease progression without clinical benefit, unacceptable treatment-related toxicities, patient request, or death. Patients were allowed to be treated beyond initial evidence of disease progression if the treating investigator determined that the patient was deriving clinical benefit from nivolumab, was tolerating study drug and had a stable performance status. Patients were allowed to receive SRS or WBRT for CNS disease progression and be continued on nivolumab, beyond progression.

At baseline and every 3 months on study, patients were followed by brain MRI, CT scan of the chest, abdomen and pelvis, and neurocognitive and QOL questionnaires. MRI’s were obtained using institutional standard of care protocols every 3 months. The MRI slice thicknesses ranged from 0.8 to 1 mm obtained on 1.5–3 T machines. Response was evaluated using RECIST version 1.1 and excluded lesions that were treated with SRS. Suspected progressions underwent repeated imaging studies after 4–6 weeks to better define the disease status and to differentiate pseudoprogression from progressions. If this subsequent imaging confirmed disease progression, the date of progression was recorded as the date of the initial scan where progression was suspected. An independent and central evaluation of brain MRI was conducted at the end of the study by a neuroradiologist (FG) to evaluate the intracranial response and rate of radionecrosis of SRS treated metastases.

QOL was assessed using the Functional Assessment of Cancer Therapy—Brain (FACT-BR), for which the range is from 0 to 200 and higher scores indicate better QOL. Neurocognitive function was assessed using a well-established battery of cognitive tests: the Hopkins Verbal Learning Test—Revised (HVLT-R: learning, immediate and delayed memory),^14^ Trail Making Test (TMT: processing speed and executive function),^15^ and the Controlled Oral Word Association (COWA: verbal fluency).^16^ The tests have published normative data that took into account age and, where appropriate, education and gender.

### Endpoints

The primary endpoint was iPFS, with death and disease progression within the brain as events. Intracranial failure was defined using the RECIST version 1.1 criteria of SRS treated and untreated brain. Secondary endpoints consisted of intracranial progression rate, extracranial progression rate, progression free survival (PFS), overall survival (OS), and change in QOL and neurocognitive function. For intracranial progression rate, events were intracranial progression and death without progression considered as competing risk. For extracranial progression rate, events were progression outside the brain and death without progression considered as competing risk. FACT-BR was scored as per the scoring template provided by FACIT (FACIT system). Cognitive deterioration was defined as a decline of greater than 1 SD from baseline on at least 1 of the 7 cognitive tests (all tests were standardized based on published norms and transformed so that higher values represent improved cognition) at the 3-, 6- and 9-month intervals post-SRS.

### Statistical Analysis

Trial accrual was closed on January 7, 2020. The data was locked on January 31, 2021 for this analysis. The study’s sample calculations suggested that 60 patients (30 patients per histology) were needed to evaluate the effect of the treatment combination in improving 1-year iPFS from historical values of 24–50%.^12,17^ With the consensus of the study investigators and data safety monitoring board, accrual was stopped at 26 patients due to the increased use of combinatorial immune checkpoint inhibitors (ICB). Per intention to treat, the iPFS for the 26 patients was estimated with 95% two-sided confidence intervals (95 CI). Time to event analyses used the date of the first nivolumab cycle as the starting time. Time to intracranial and extracranial progression rates were analyzed using Cox proportional hazards and competing risks models, with death as a competing risk. PFS was defined as the time from the first cycle until the date of first progression (either intracranial or extracranial progression whichever occurred first) or death date or last follow-up whereas OS events were the dates of deaths and last follow-up. Survival free of neurocognitive decline was the time to the first 1 SD deterioration of any neurocognitive test or death from baseline test date among patients who completed the neurocognitive tests at 3 months.^3^ Changes in QOL and neurocognitive scores from baseline were compared using a student paired *t*-test and repeated measures analysis of variance (R-ANOVA). All secondary analyses used a two-sided .05 level of significance. The R package (version 3.6.3; R Foundation for Statistical Computing, Vienna, Austria; https://www.R-project.org/) was used primarily for statistical analysis.

This study is registered with Clinicaltrials.gov, NCT02978404.

## Results

### Patient Characteristics

**Figure 1.**
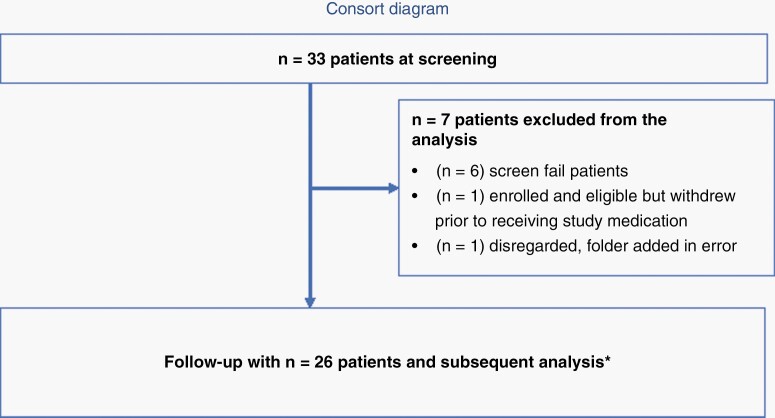
Protocol enrollment and analysis diagram. ^*^3 patients did not undergo radiosurgery and 3 patients did not undergo a follow-up MRI at 3 months, and were not included in the central MRI review of intracranial disease.

From August 2, 2017 to January 3, 2020, 33 patients were screened of which 22 NSCLC and 4 RCC patients were treated under the study protocol (Figure 1). Fifteen patients received nivolumab as first line systemic treatment. Nine (34.6%) patients were previously treated with local CNS therapy. The median number of BM treated was 3 (range 1–9 lesions) among the 23 patients who proceeded to receive SRS. The median Graded Prognostic Assessment (GPA) score of the patients was 2 (range: 1–3). Baseline patient characteristics are shown in Table 1. The median follow-up was 15.0 months (range: 0.43–25.9 months).

**Table 1.** Patient and disease characteristics

Gender	
Female:Male	13:13
Age	
Median (min, max)	63.5 (46, 84)
ECOG	
0	17
1	8
2	1
Cancer histology	
NSCLC	22 19 Adenocarcinoma 2 Adenosquamous 1 squamous
RCC (clear-cell)	4
Tumor PD-L1 status	
Unknown	5 (4 RCC, 1 insufficient material)
<1%	5
1–49%	4
≥50%	12
Prior systemic therapy	
0 Line	15
1 Line	4
2–5 Lines	7
Prior brain metastasis treatments	
Yes	9
SRS treated lesions	
Median (min, max)	3 (1, 9)
Total SRS volume (cc)	
Median (min, max)	0.78 (0.078, 6.4)
Presence of extracranial disease	
Yes	22
Follow-up	
Median months (min, max)	15.0 (0.43, 25.9)

NSCLC, non-small cell lung carcinoma; RCC, renal cell carcinoma.

### Treatment Response

**Figure 2.**
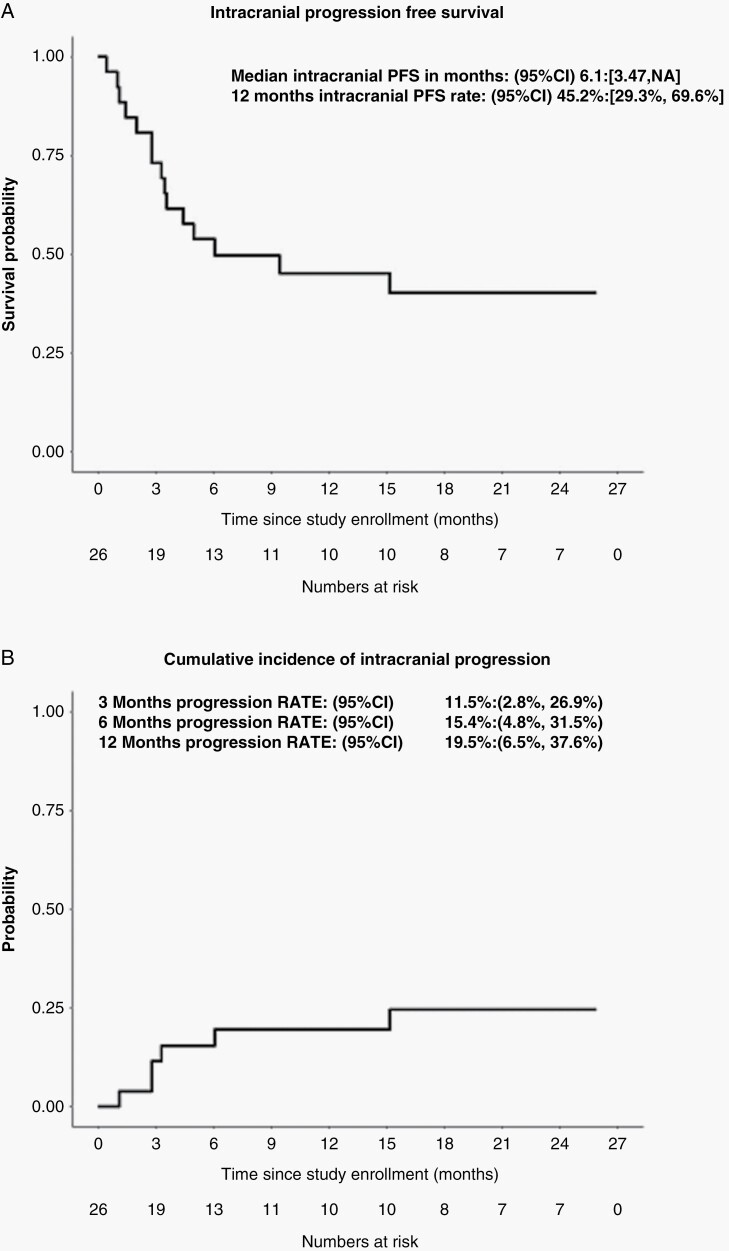
Patient intracranial disease control. (A) Kaplan–Meier curve of intracranial progression free survival (iPFS). The 95% confidence interval (95% CI) of the median and 12-month rate estimate is indicated. (B) Cumulative intracranial progression probability and 95% confidence interval (95% CI). Estimate was made using competing risk analyses with death without progression considered as competing risk.

**Figure 3.**
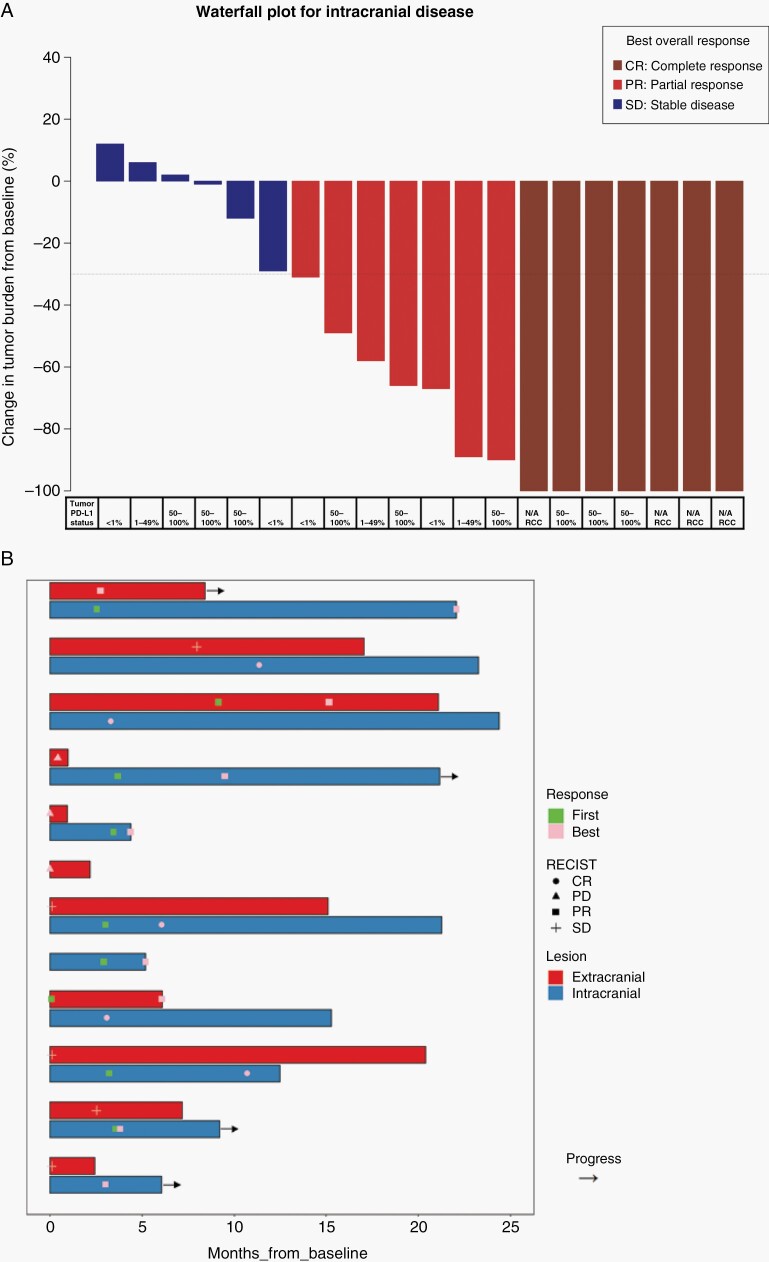
Best overall response waterfall plots. (A) Maximum percent change in the aggregate diameter of Response Evaluation Criteria in Solid Tumors (RECIST) of intracranial metastases. Tumor PD-L1 status (PD-L1 ICH 22c3 pharmDX assay) are indicated underneath. (B) Time to and duration of response in brain and extracerebral lesions for patients who had a brain metastasis response or remained on trial for at least 3 months (11 of 20 evaluable patients). Bars are grouped by the same patient’s brain and extracranial response.

One-year iPFS was 45.2% (95 CI: 29.3–69.6%) (Figure 2A) and median iPFS was 6.1 months (95 CI: 3.5–NA months). Using death as a competing risk, the cumulative intracranial progression probability was 19.5% (95 CI: 6.5–37.6%) at 1 year (Figure 2B). Of the patients that developed intracranial progressions, 1 patient failed within SRS treated lesions while the remaining intracranial failures were new BM. Of the 4 lesions treated using 15 Gy, this local failure occurred in a 2.4 cc left-frontal lesion. Overall response (partial or complete) of SRS treated lesions was seen in 14 out of the 20 patients with at least 1 evaluable follow-up MRI (Figure 3A). By 3 months, 11 patients had already achieved an intracranial response (Figure 3B). There was a significant mean reduction in the diameter of 76 evaluable SRS treated lesions as compared to baseline (−38.7%; *P* = .001).

**Figure 4.**
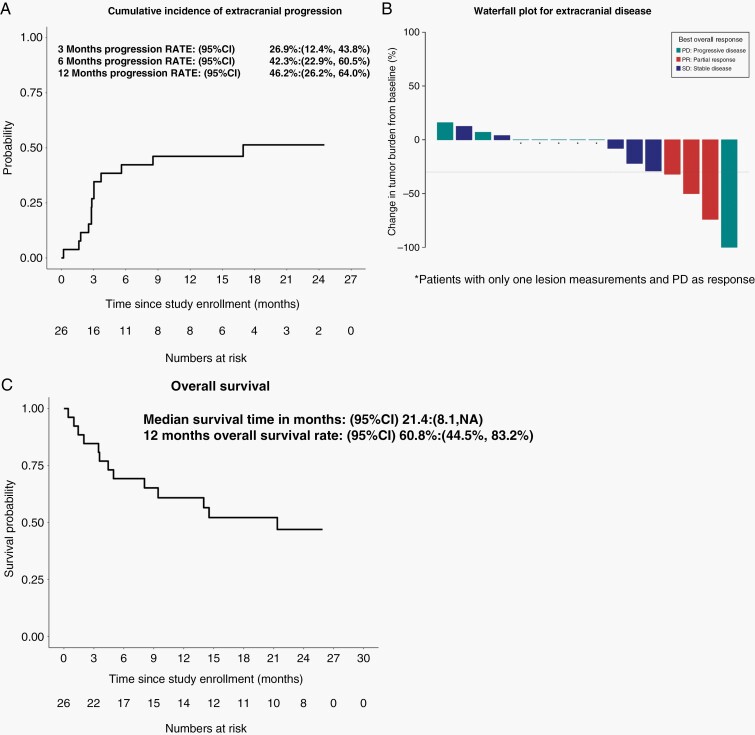
Patient overall survival and extracranial disease control. (A) Kaplan–Meier curve of overall survival and (B) Cumulative extracranial progression probability estimated using competing risk analyses with death without progression considered as competing risk. The 95% confidence intervals (95% CI) of the medians and 12-month rate estimates are indicated. (C) Maximum percent change in aggregate diameter of un-irradiated RECIST extracranial target metastases. RECIST response are color coded to include non-target lesion response.

The median OS of the patients was 21.4 months (95 CI: 8.1–N/A) (Figure 4A). OS was not correlated (*P* = .39) with tumor PD-L1 status or histology. Five out the 26 patients had no extracranial RECIST evaluable disease so extracranial response was assessable in 21 patients only. Among the 21 patients with RECIST evaluable disease, extracranial progression occurred in 13 patients as the first event of progression while 5 patients died prior to completing their first response evaluation. Four patients (15%) ­attained a partial response as their best response for extracranial lesions (Figure 4B). Using death as a competing risk analysis, the cumulative extracranial progression probability at 6 month and 12 months were 42.3% (22.9–60.5%) and 46.2% (26.2–64.0%) following nivolumab initiation (Figure 4C).

### Toxicity

AEs secondary to the nivolumab were comparable to previous studies involving PD-1 inhibitors (Supplementary material A). Two patients had Grade 3 fatigue attributable to SRS and nivolumab. Two patients required steroid treatments after radiosurgery. One patient died of a myocardial infarction that was unrelated to the study interventions. One (4%) patient was suspected to have an SRS induced radionecrotic lesion, which on subsequent MRIs continued to grow over the year and on central review was consistent with disease progression. Thus, there was no radionecrosis suggesting that 95CI of patients developing radionecrosis is 0–13%.

### Patient Reported Outcomes

Neurocognitive evaluation was completed by 16 and 9 patients at 3 and 6-month follow-up, respectively. Median age of the 16 patients was 63 (range: 46–77). There was no significant difference in the patient’s mean performance in any of the neurocognitive assessments from baseline to 3-month follow-up. A deterioration of ≥1 SD in at least 1 assessment from baseline was observed in 8 (50%) patients at 3 months. At 6 months, participants significantly improved on the TMT- Parts A (*P* = .04) and Parts B (*P* = .03) (Supplementary material B). The rate of survival free of neurocognitive decline was 73.7% (95 CI: 54.5–99.6%) at 3 months.

**Figure 5.**
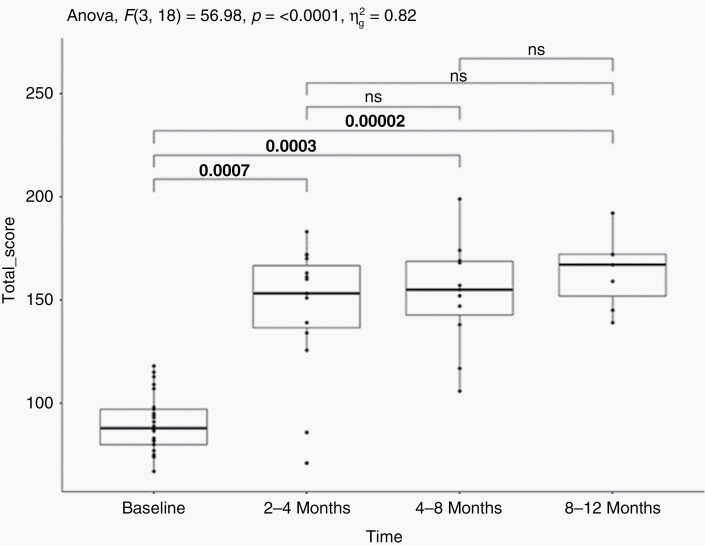
Total FACT-BR score over the course of time. Higher values represent improved quality of life. The overall trend over time is significant (Repeated ANOVA *P* < .0001). Pair-wise *t*-test with Bonferroni adjusted *P*-values are also indicated with brackets. ns, Non-significant.

QOL questionnaires were completed by 16, 11 and 7 patients within 2–4 months, 4–8 months and 8–12 months from treatment initiation, respectively. The mean FACT-Br total scores were 90.2 (SD 13.8), 146.2 (SD 31.6), 152.9 (SD 26.1) and 163.7 (SD 17.9) at baseline, 2–4 months, 4–8 months and 8–12 months from treatment initiation, respectively. Mean FACT-Br total scores were significantly (*P* < .0001, repeated measures ANOVA) better at all time points than baseline (Figure 5).

## Discussion

ICBs, especially inhibitors of the PD-1/PD-L1 pathway have an increasing role in the treatment of malignancies, including NSCLC and RCC. Improvements in local and systemic therapies have led to improved life expectancy and a subsequent increased risk of developing BM from tumors, including NSCLC and RCC.^9,17–19^ Following SRS treatment alone of small BM, 55–78% of patients will develop new BM^2–5^ within 1 year. Although SRS incurs less cognitive side effects than WBRT, it can lead to vasogenic edema and radionecrosis that necessitate long-term steroids, which impede the efficacy of ICBs.^18^ Upfront initiation of SRS and ICB could improve BM control and this current study aimed to evaluate the safety and efficacy of concurrent nivolumab and SRS in patients with BM from NSCLC and RCC.

Of the 26 study subjects, 23 patients underwent SRS as 3 patients did not undergo SRS due to rapid progression of their disease from study entry and SRS was no longer indicated. Beyond AEs secondary to nivolumab, we observed two grade 3 fatigues in two patients that were attributable to SRS. Two patients needed a new prescription or an increased dose of dexamethasone to control for AEs (headache and SRS related brain edema). Following central review of the brain MRIs, none of the patients developed brain radionecrosis in the lesions treated under the study. The relative safety of the study regimen was further supported by a significant improvement in patient QOL from baseline to 2–4 months after study initiation [mean FACT-Br total scores: 90.2 (SD 13.8) vs. 146.2 (SD 31.6; *P* = .0007)]. This QOL improvement from baseline was maintained (*P* < .0001) within the subsequent year among patients who continued to respond to the questionnaires.

The 1-year iPFS of the study patients was 45.2% (95 CI: 29.3–69.6%). The 1-year cumulative intracranial recurrence probability was estimated to be 19.5% (95 CI: 6.5–37.6%). Compared to the previously described studies evaluating the treatment of SRS only,^2–5^ the current regimen improved the 1-year iPFS from 16–27% to 45.2% and reduced the 1-year intracranial recurrence from 55–78% to 19.5%. Indeed, even though the current study enrolled patients with >3 BM and those previously treated for prior BM using local techniques, the intracranial recurrence rate from the current regimen was comparable to the 1-year intracranial control (15.0%) observed by Brown et al^3^ by adding WBRT to SRS in the treatment of patients with 1–3 BM not previously treated for BM. Furthermore, the rate of neurocognitive deterioration from the current study’s patients was similar to patients undergoing SRS only. Using the same methodologies, Brown et al. documented that 63.5% and 91.7% of the respondent patients had cognitive declines in SRS and SRS + WBRT arms respectively at 3 month, whereas the current study observed that 50% of the patients had cognitive decline at 3 months. Thus, the current method in combining nivolumab with SRS improved the intracranial disease control as WBRT would, without inflicting the neurocognitive side effects from WBRT.

Reports from Goldberg et al, Long et al, and Flippot et al examined the use of anti-PD-1/PD-L1 in the treatment of BM from melanoma, NSCLC and RCC.^9–11^ The authors reported overall intracranial response rates of 12–29.7 % from anti-PD-1/PD-L1 monotherapy. Specifically, in asymptomatic patients, Goldberg et al^9^ reported median iPFS of 2.3 months among NSCLC patients who had ≥1% PD-L1 expression and Flippot et al^11^ reported median iPFS of 2.7 months among RCC patients who had previously untreated BM. After pembrolizumab monotherapy for ≥1% PD-L1 expressing NSCLC, 67% of the patients (*n* = 27) progressed in the CNS within 1 year.^9^ In RCC, Flippot et al. observed that 72% of the patients needed focal brain therapy (36% SRS, 23% WBRT ± SRS, 13% surgery ± SRS) following nivolumab. Furthermore, 49% of the patients developed symptoms related to intracranial disease and 50% required corticosteroid use. It can be concluded from the above studies that despite observing intracranial activity of anti-PD-1/PD-L1, most patients progressed rapidly within the CNS and needed local therapy or WBRT. The current study observed a median iPFS of 6.1 months (95 CI: 3.5–NA), which represented a clinically meaningful improvement in controlling the disease from progressing in the brain. Of the 20 patients with centrally reviewed brain MRIs, the overall intracranial response rate was 70% (7 PR, 7 CR) with important (≥30%) reduction in diameter of BM (mean −38.7%) by 3 months. Tumor PD-L1 expression did not seem to affect the response of BM. The combination of SRS with nivolumab may improve the local effect of SRS as among the 76 evaluable lesions treated with SRS, only 1 local failure occurred. This compares favorably with the 18.2% and 27.4% local failures observed at 6- and 12-months following SRS only in the study from Brown et al.^19^ An SRS dose-response relationship could not be assessed because only 1 local failure was observed among SRS treated lesions.

From a multi-institutional database of 6984 patients newly diagnosed with BM from 2006 to -2017, Sperduto et al^20^ developed a robust diagnostic GPA to estimate OS. This GPA system would estimate the median OS of our cohort to be 12.5 months. Also, Goldberg et al^9^ observed a median OS of 9.9 months in patients receiving pembrolizumab monotherapy for BM from ≥1% PD-L1 expressing NSCLC. Previous retrospective and prospective registry studies sought to characterize the role of radiotherapy to BM in combination with immunotherapy, which included various radiation regimens at various time intervals and sequence from different forms of immunotherapy.^21–27^ These studies, involving mostly melanoma patients, suggested that the addition of radiotherapy, and in particular SRS to ICBs may improve OS. Further subgroup analyses suggested that the benefit of RT is mainly in patients treated concurrently (within 4 weeks) from the initiation of ICB. Patients who received RT prior to ICB or at the time of intracranial failure during ICB had inferior outcomes. Thus, this trial sought to evaluate a uniform treatment strategy in which 1 dose of SRS was given within 14 days of the first Nivolumab infusion. The improved median OS from this study’s patients of 21.4 months, was likely associated with the combined effects of ICB and SRS in improving intracranial disease.

The probability of extracranial progression remains high at 46.2% at 1 year (13 events), which along with death (8 events) were the leading cause of treatment discontinuation. Continued improvements in ICB development will lengthen patient survival. For example, the Checkmate 9LA trial observed a further improvement in median OS (20.8 months) through the addition of two cycles of chemotherapy prior to ipilimumab/nivolumab in patients with BM.^28^ Testing the combination of SRS with more aggressive immunotherapy, Li et al^29^ presented encouraging tolerability data when SRS was combined with ipilimumab/nivolumab. Alternatively, given the good tolerability of SRS + nivolumab, this combination could be started upfront, prior to adding ipilimumab or ipilimumab/chemotherapy in subsequent cycles to improve patient adherence within this more fragile population.

In this study, no clinical or radiological radionecrosis was observed. Initial retrospective studies suggested that immune checkpoint inhibition increased the rate of radionecrosis from SRS. For example, Martin et al and Colaco et al, both observed an increased incidence of radionecrosis in patients who received ICI during SRS 36–37.5%, which consisted of mostly melanoma patients receiving a variety of ICIs at various interval from SRS.^30,31^ However, the current study’s observation was more consistent with more recent reports from Trommer et al and Lehrer et al, in which radionecrosis was seen in 4.3–11.7% of the patients.^21–25^ Larger prospective trials are needed to better characterize the risk or radionecrosis based on the cancer type and SRS-ICI combination strategy.

The current study was limited by the small and heterogeneous study population, mainly consisting of NSCLC. Accrual to this study spanned an era during the transition from chemotherapy to immune checkpoint inhibition leading to 42% of the patients receiving study nivolumab as second- or third-line systemic treatment. The shifting landscape in the systemic treatment of NSCLC and RCC also slowed patient recruitment. Trial accrual was stopped early in response to the increased use of combinatorial ICB’s instead of anti-PD-1 monotherapy in metastatic NSCLC and RCC. Despite the limitations, this multi-institutional prospective phase II study was one of the first studies to evaluate the safety and efficacy in combining SRS with nivolumab for patients with BM. Patient QOL and neurocognitive functions were captured to support the safety, tolerability and long-term benefit of this combinatorial strategy. Central review of brain MRIs were carried out to document and scrutinize the response and rate of radionecrosis from SRS given during nivolumab.

The current trial supported ASCO and FDA’s statements in including more patients with BM in ongoing trials.^32,33^ Our study suggested that treating BM early achieved better intracranial control, which could reduce subsequent treatment interruptions and steroid use. Further validation study of concurrent ICB with SRS for brain metastases appears justified.

## Supplementary Material

vdad018_suppl_Supplementary_Appendix_S1

vdad018_suppl_Supplementary_Appendix_S2
