# Supplementary material for: Phase II multicenter trial combining nivolumab and radiosurgery for NSCLC and RCC brain metastases
Source: Neurooncol Adv. 2023 Mar 1;5(1):vdad018. doi: 10.1093/noajnl/vdad018 (PMC10072191; doi:10.1093/noajnl/vdad018)

1. Adverse events (AE) (1) irrespective of relationship with treatments, (2) serious AEs attributed (possible, probable and definitely related) to nivolumab, (3) Grade 3+ and serious AEs attributed (possible, probable and definitely related) to nivolumab and radiosurgery and (4) the median (range) of months in the development of treatment related AEs in relation to the first administration of nivolumab and radiosurgery.

1)

|  | **Patients N (%) regardless of attribution** | |
| --- | --- | --- |
| **AE type** | Any grade | Grade 3-5 |
| **Respiratory** | **22 (85%)** | **2 (8%)** |
| **General** | **20 (77%)** | **3 (12%)** |
| **Gastrointestinal** | **19 (73%)** | **2 (8%)** |
| **Musculoskeletal** | **19 (73%)** | **2 (8%)** |
| **Nervous system disorders** | **17 (65%)** | **5 (19%)** |
| **Infections** | **14 (54%)** | **2 (8%)** |
| **Skin** | **13 (50%)** | **1 (4%)** |
| **Other (Psychiatric, Lab, Immune system, Blood and lymphatic system, Metabolism and nutrition , Cardiac, Eye, Ear and labyrinth, Endocrine, Renal and urinary, Vascular, Injury, procedural complications)** | **22 (85%)** | **6 (23%)**  **(one G5 myocardial infarction)** |

2)

| **AE Term** | **GRADE** | **Attribution to nivolumab** | **AE Type** |
| --- | --- | --- | --- |
| **LEFT HIP PAIN** | **2** | **Possible** | **Musculoskeletal and connective tissue disorders** |
| **COLITIS** | **3** | **Probable** | **Gastrointestinal disorders** |
| **ASEPTIC MENINGITIS** | **3** | **Possible** | **Infections and infestations** |
| **FATIGUE** | **3** | **Possible** | **General disorders and administration site conditions** |
| **NEPHRITIS** | **3** | **Probable** | **Renal and urinary disorders** |

3)

| **AE Term** | **GRADE** | **Attribution to Nivo** | **Attribution to SRS** | **AE Type** | **Serious** |
| --- | --- | --- | --- | --- | --- |
| **FATIGUE** | **3** | **Possible** | **Probable** | **General disorders and administration site conditions** | **Yes** |
| **FATIGUE** | **3** | **Definite** | **Possible** | **General disorders and administration site conditions** | **No** |

4)


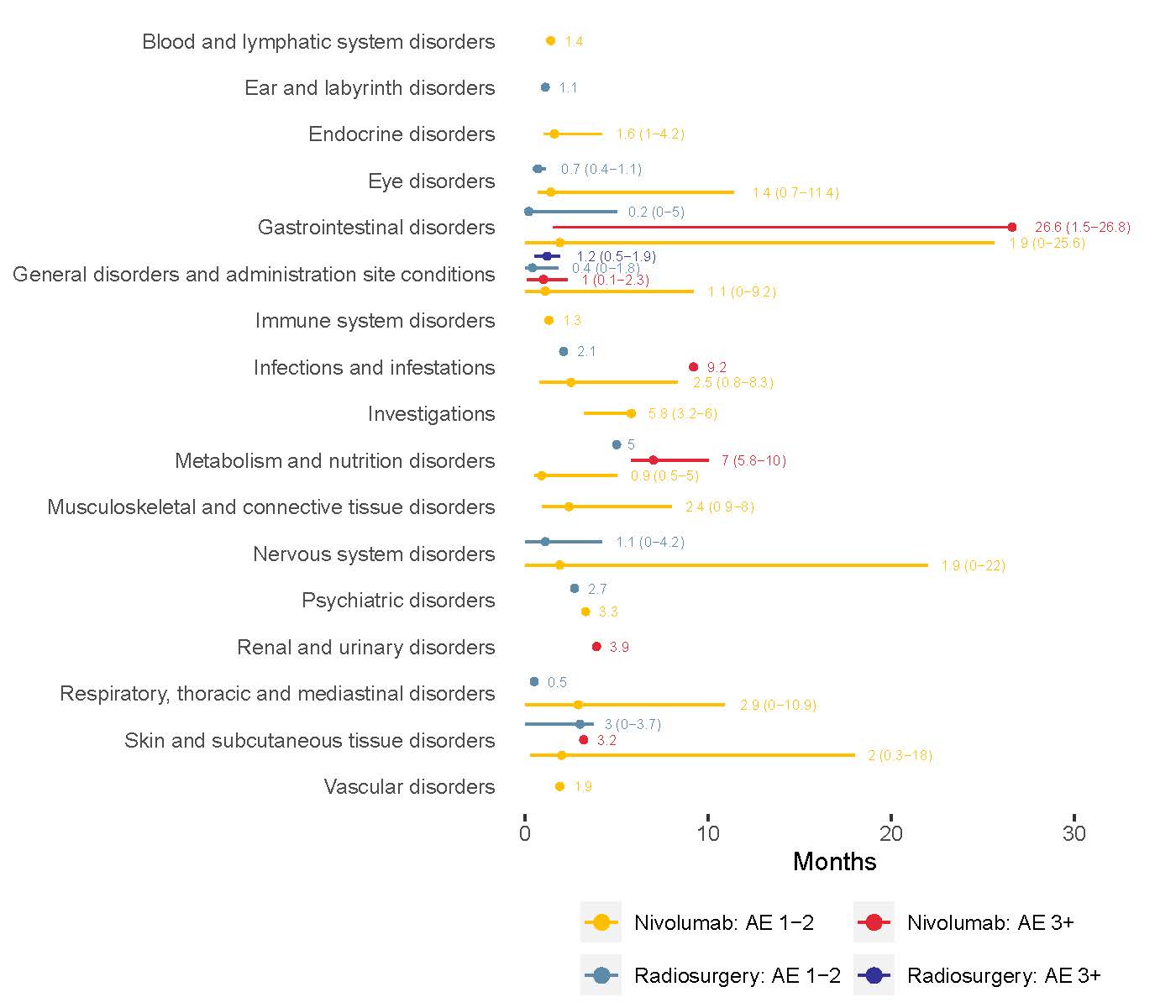

Supplement: vdad018_suppl_Supplementary_Appendix_S1 [file vdad018_suppl_supplementary_appendix_s1.docx]
