# Supplementary figures and images for: Phase II multicenter trial combining nivolumab and radiosurgery for NSCLC and RCC brain metastases
Source: Neurooncol Adv. 2023 Mar 1;5(1):vdad018. doi: 10.1093/noajnl/vdad018 (PMC10072191; doi:10.1093/noajnl/vdad018)

**B.** Neurocognitive performance at 3 (1) and 6 months (2)

1)


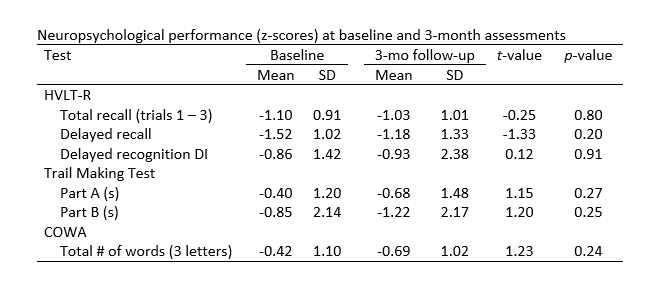


2)


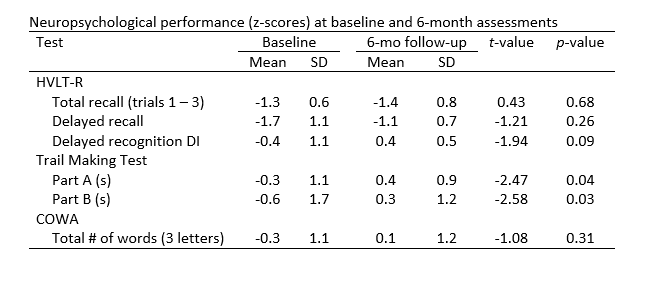

Supplement: vdad018_suppl_Supplementary_Appendix_S2 [file vdad018_suppl_supplementary_appendix_s2.docx]
